# Lattice strain-enhanced exsolution of nanoparticles in thin films

**DOI:** 10.1038/s41467-019-09395-4

**Published:** 2019-04-01

**Authors:** Hyeon Han, Jucheol Park, Sang Yeol Nam, Kun Joong Kim, Gyeong Man Choi, Stuart S. P. Parkin, Hyun Myung Jang, John T. S. Irvine

**Affiliations:** 1 Department of Materials Science and Engineering, Pohang University of Science and Technology (POSTECH), Pohang, 37673 Republic of Korea; 2 Max Planck Institute of Microstructure Physics, Weinberg 2, Halle (Saale), 06120 Germany; 3 Gyeongbuk Science & Technology Promotion Center, Gumi Electronics & Information Technology Research Institute, Gumi, 39171 Republic of Korea; 4 Department of Materials Science and Engineering, Kumoh National Institute of Technology, Gumi, 39177 Republic of Korea; 5 1Fcell Inc., Pohang, 37673 Republic of Korea; 6 School of Chemistry, University of St Andrews, St Andrews, KY16 9ST Scotland UK; 7 Present Address: Department of Materials Science and Engineering, Massachusetts Institute of Technology, Cambridge, MA 02139 USA; 8 Present Address: Research Institute of Advanced Materials, Seoul National University, Seoul, 08826 Republic of Korea

**Keywords:** Catalyst synthesis, Electrocatalysis

## Abstract

Nanoparticles formed on oxide surfaces are of key importance in many fields such as catalysis and renewable energy. Here, we control B-site exsolution via lattice strain to achieve a high degree of exsolution of nanoparticles in perovskite thin films: more than 1100 particles μm^−2^ with a particle size as small as ~5 nm can be achieved via strain control. Compressive-strained films show a larger number of exsolved particles as compared with tensile-strained films. Moreover, the strain-enhanced in situ growth of nanoparticles offers high thermal stability and coking resistance, a low reduction temperature (550 °C), rapid release of particles, and wide tunability. The mechanism of lattice strain-enhanced exsolution is illuminated by thermodynamic and kinetic aspects, emphasizing the unique role of the misfit-strain relaxation energy. This study provides critical insights not only into the design of new forms of nanostructures but also to applications ranging from catalysis, energy conversion/storage, nano-composites, nano-magnetism, to nano-optics.

## Abstract (web summary)

Dispersion of metallic nanoparticles is promising for energy conversion and storage, but gaining control of size and distribution is not trivial. Here the authors use lattice mismatch to manipulate exsolution of nanoparticles, achieving a high population of small nanoparticles in perovskite thin films.

## Introduction

Nanoscale functional materials have generated a broad interest in many fields that include electronics, optics, magnetism, superconductivity, and catalysis due to the possibilities of generating novel physical phenomena. Metal nanoparticles dispersed on oxide surfaces have been extensively investigated due to their key role in catalysis, energy conversion, and energy storage, including batteries, fuel cells, and electrolysis cells^1–4^. These metal particles have mainly been prepared by deposition techniques. However, the deposited particles mostly show limitations in particle size and distribution control, as well as degraded properties resulting from agglomeration or carbon coking^5–7^. The exsolution of the B-site ions from perovskite lattices (ABO_3_) under reducing conditions is emerging as an alternative technique to allow for the possibility of the in situ growth of nanoparticles. Compared to deposition methods, this process shows better cost- or time-efficiency, good thermal stability, and resistance to coking problems^8–13^. However, the exsolution from stoichiometric ABO_3_ perovskites has shown (i) a limited number of active cations, (ii) a preference for formation within the bulk rather than at or on the surface, and (iii) a slow speed of particle generation^14–16^. To overcome these deficiencies, A-site deficient perovskites were employed to promote B-site cation exsolution on surfaces, leading to both A-site and oxygen deficiencies that allow for more rapid ion diffusion and electron generation during reduction by hydrogen^10–12^. It was further revealed that voltage-driven reduction is two orders of magnitude faster than conventional reduction and yields a small particle size (~15 nm) with a population density as high as ~400 particles μm^−2^, resulting in outstanding electrochemical activity^13^.

To date, the studies of such exsolution processes have been performed predominantly in bulk polycrystalline ceramics. Unlike bulk systems, thin-film heterostructures can induce a lattice strain because of the lattice mismatch between a substrate and a film, which affects many physical properties such as ferroelectricity, electron mobility, ionic conductivity, and electrocatalysis^17–20^. In particular, thin-film oxide fuel cells are attracting renewed attention owing to advantages of low temperature operation and portable device applications^21–24^. Here, we demonstrate an unprecedently high degree of exsolution of nanoparticles in lattice misfit strained epitaxial thin films and achieve a particle density as high as ~1100 particles μm^−2^, with a size of only ~5 nm, at a temperature as low as 550 °C. Compressive-strained films show a larger number of exsolved metal particles than tensile-strained films. Furthermore, we have demonstrated a wide tunability of particle growth in strained films. The mechanism of the lattice strain-enhanced exsolution has been revealed by thermodynamic and kinetic models. Manipulation of nanoparticle structures using these concepts can be further applied to develop nano-composite functional films^25, 26^, nano-phase magnetic materials^27–29^, and nano-optics^30^.

## Results

### Structural change via exsolution of nanoparticles in strained thin films

**Fig. 1:**
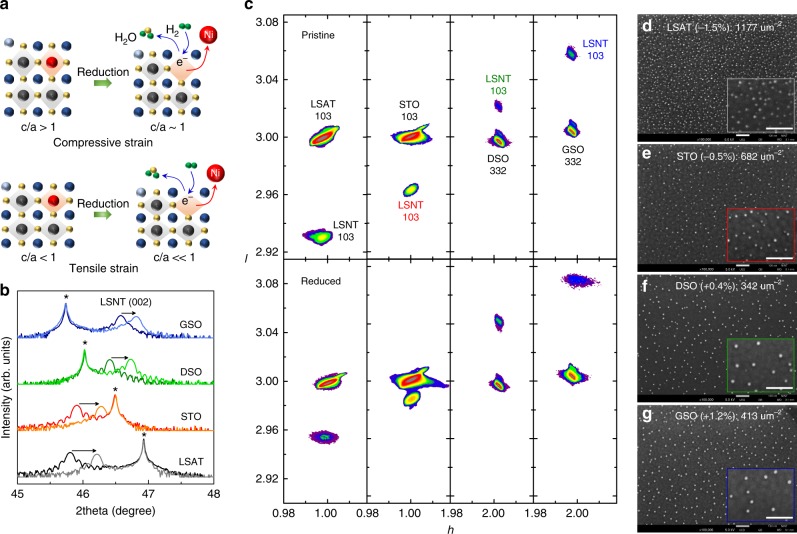
Strain-enhanced exsolution in thin films. **a** Schematic representation of the reduction process in compressive- and tensile-strained thin films, where light blue circles denote La ions, blue circles denote Sr ions, red circles for Ni ions, black circles for Ti ions, yellow circles for O ions, and green circles designate H ions. **b** *θ* − 2*θ* XRD patterns of pristine and reduced LSNT films PLD-grown on four different substrates. **c** Corresponding RSM studies about 103-diffraction conditions of the pristine and reduced films grown on four different substrates. SEM micrographs of the reduced (pure H_2_, 550 °C, 80 h) LSNT films on **d** LSAT, **e** STO, **f** DSO, and **g** GSO substrates, respectively. Scale bars, 100 nm

Epitaxial thin films (100-nm-thin) were deposited on four distinct lattice mismatched substrates via pulsed laser deposition (PLD). The substrates chosen are (LaAlO_3_)_0.3_–(SrAl_0.5_Ta_0.5_O_3_)_0.7_ (LSAT) (001), SrTiO_3_ (STO) (001), DyScO_3_ (DSO) (110), and GdScO_3_ (GSO) (110), having (pseudo-)cubic lattice parameters of 3.868, 3.905, 3.944, and 3.973 Å, respectively. Thin films were grown on these substrates using a La_0.2_Sr_0.7_Ni_0.1_Ti_0.9_O_3−*δ*_ (LSNT) target having a 10% A-site deficiency. The PLD-fabricated pristine LSNT films were then reduced in a furnace by flowing dry H_2_ at 550 °C for 80 h. Figure 1a schematically depicts the exsolution process of Ni particles in the strained films. Owing to the reducing atmosphere, oxygen ions are stripped from the oxide lattice, resulting in electron carriers as follows^10, 13^:

1 $$O_O^X \to V_O^{ \cdot \cdot } + 2e^ - + \frac{1}{2}O_2$$

The electrons resulting from this process lower the oxidation state of Ti, according to e^−^ + Ti^4+^ → Ti^3+^. This, along with the loss of oxygen, results in a lattice-volume expansion. The concomitant nucleation of Ni metal via 2e^−^ + Ni^2+^ → Ni^0^ will partially reverse this expansion, as it effectively removes vacancies, resulting in a volume contraction. As the reduction proceeds, the growth of Ni nuclei leads to exsolved particles on the surface. To measure the structural changes of the oxide lattice, *θ* − 2*θ* X-ray diffraction (XRD) (Fig. 1b) and reciprocal space mapping (RSM) (Fig. 1c) was carried out. The detailed results of the substrate-dependent strain and structural parameters are presented in Supplementary Figure 1. The lattice misfit strain (*ε*) of the pristine films is −1.5%, −0.5%, +0.4%, and +1.2% for LSAT, STO, DSO, and GSO substrates, respectively. After the reduction, all four LSNT films reveal a decrease in the out-of-plane (OOP) lattice parameter but show a negligible change in the in-plane lattice parameters owing to the substrate-clamping effect. Interestingly, the compressive-strained films show a reduced tetragonality (*c*/*a* ~1), returning to a stable bulk cubic-like structure after reduction. In contrast, for the tensile-strained films, the tetragonality moves away from 1 (*c*/*a* « 1) and the films become unstable after reduction (Supplementary Figure 1b). It is generally known that the exsolution in bulk ceramic systems is accompanied by an increase in the lattice volume^11–13^. On the contrary, our structural analysis based on XRD results shows that strained thin-film heterostructures yield a contraction in the lattice volume (Supplementary Figure 1b). This implies that the reduction of Ni and subsequent exsolution of Ni^0^ dominates over the Ti-reduction in the strained LSNT films.

### Effect of lattice strain on exsolution of nanoparticles

**Fig. 2:**
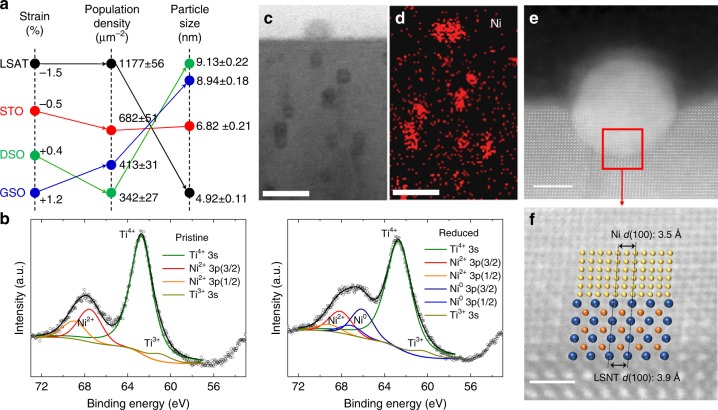
Characteristics of the exsolution in thin films. **a** Effects of the substrate on three important physical parameters related to the exsolved thin films. **b** Ni 3*p*, Ti 3*s* XPS of pristine and reduced LSNT films grown on LSAT substrates. **c** Ex-situ cross-sectional STEM-HAADF image and **d** corresponding Ni EDS map of the reduced film on LSAT substrate. The reduction was performed at 550 °C for 80 h in pure H_2_. Scale bars, 10 nm. **e** Cross-sectional bright-field STEM image of a Ni particle exsolved on (100) perovskite surface after reducing at 900 °C for 12 h in pure H_2_. Scale bar, 5 nm. **f** Magnified STEM image at the metal-perovskite interface, where turquoise circles denote La or Sr ions and orange circles denote Ni or Ti ions in the perovskite lattice, and yellow circles denote Ni ions in exsolved particles. Scale bar, 1 nm

To explore the effects of lattice strain on the extent of Ni-particle exsolution, scanning electron microscopy (SEM) analysis (Fig. 1d–g) was conducted for the reduced films. These micrographs clearly reveal that the population density of exsolved particles on the surface is greatly affected by the lattice strain. Figure 2a compares various physical characteristics of the films to help understand the difference in population density and size of the exsolved particles. The population density is sensitive to the sign and magnitude of the lattice strain and increases according to the following sequence of substrates**:** DSO (*ε* = +0.4%), GSO (*ε* = +1.2%), STO (*ε* = −0.5%), and LSAT (*ε* = −1.5%) substrates. On the other hand, the exsolved particle size has an opposite tendency with the smallest size observed for LSAT. These results indicate that compressive-strained films produce a significantly larger number of exsolved particles with a smaller particle size than tensile-strained films. As described previously, the compressive-strained film transforms back towards the stable bulk cubic structure after exsolution (*c*/*a* ~1). In contrast, the tensile-strained film evolves towards a more highly distorted and unstable state after the exsolution, yielding *c*/*a* « 1 (Fig. 1a). The chemical potential change (∆*μ*) of the compressive-strained film during the reduction is negative (i.e., spontaneous) yielding a cubic-like structure with *c*/*a* ~1, while ∆*μ* of the tensile-strained film is positive (thermodynamically unfavorable) resulting in *c*/*a* « 1 after the reduction (Fig. 2a). The thermodynamic structural stability is likely the cause of the higher population density in the compressive-strained films. For a given strain type, the exsolved particle population (density) is well correlated with the magnitude (i.e., absolute value) of strain. In contrast, the particle size tends to decrease with the absolute strain value. Thus, the LSNT film grown on the LSAT substrate is highly compressive-strained with *ε* = −1.5% and yields the highest population density of exsolved particles (1177 particles μm^−2^) and the smallest particle size (4.92 nm). This can be viewed as the best candidate for catalytic nanoparticles due to the high surface coverage. These results are very impressive when compared with the previous best results obtained by voltage-driven exsolution from bulk materials, for which a population density of ~400 particles μm^−2^ with a particle size of ~15 nm has been reported^13^.

According to XPS analysis (Fig. 2b and Supplementary Figure 2), a significant amount of exsolved Ni^0^ appears after reduction, with the Ni^0^ fraction (as compared to the total Ni in the LSNT film) ranging from ~49% to ~56% depending on the substrate used. In contrast to this observation, the fraction of Ti^3+^ ions does not show a substantial change even after reduction. These XPS results clearly support our previous assumption based on analysis of the XRD lattice-volume changes.

To further examine the strain-dependent exsolution, scanning transmission electron microscopy (STEM) analysis and high-angle annular-dark-field (HAADF) TEM studies were carried out on the reduced films (Fig. 2c, d and Supplementary Figure 3). The SAED patterns (Supplementary Figure 3c, d) reveal that the deduced *c*/*a* ratios accord well with the estimated tetragonality (*c*/*a*) obtained from the XRD data (Supplementary Figure 1b). The STEM images and the corresponding EDS results (Fig. 2c, d, Supplementary Figure 3e-j, and Supplementary Figure 4g-i), show that Ni clusters are found on the surface and also suggest that some may also be found in the interior region. Notably, a bright-field STEM image of the socketed particle (900 °C, 12 h, dry H_2_) shows a ~50% submergence into the perovskite surface (Fig. 2e), implying a strong particle–substrate interaction and high resistance to agglomeration and coking. Interestingly, despite a large lattice mismatch between the exsolved particles (3.5 Å) and perovskite lattice (3.9 Å), they show a well-defined crystal orientation relationship at the interface (Fig. 2f). The atomic *d*-spacing of the exsolved particle coincides with the lattice parameter of Ni (3.5 Å), which is distinct from that of NiO (4.2 Å).

### Tunable exsolution in thin films

**Fig. 3:**
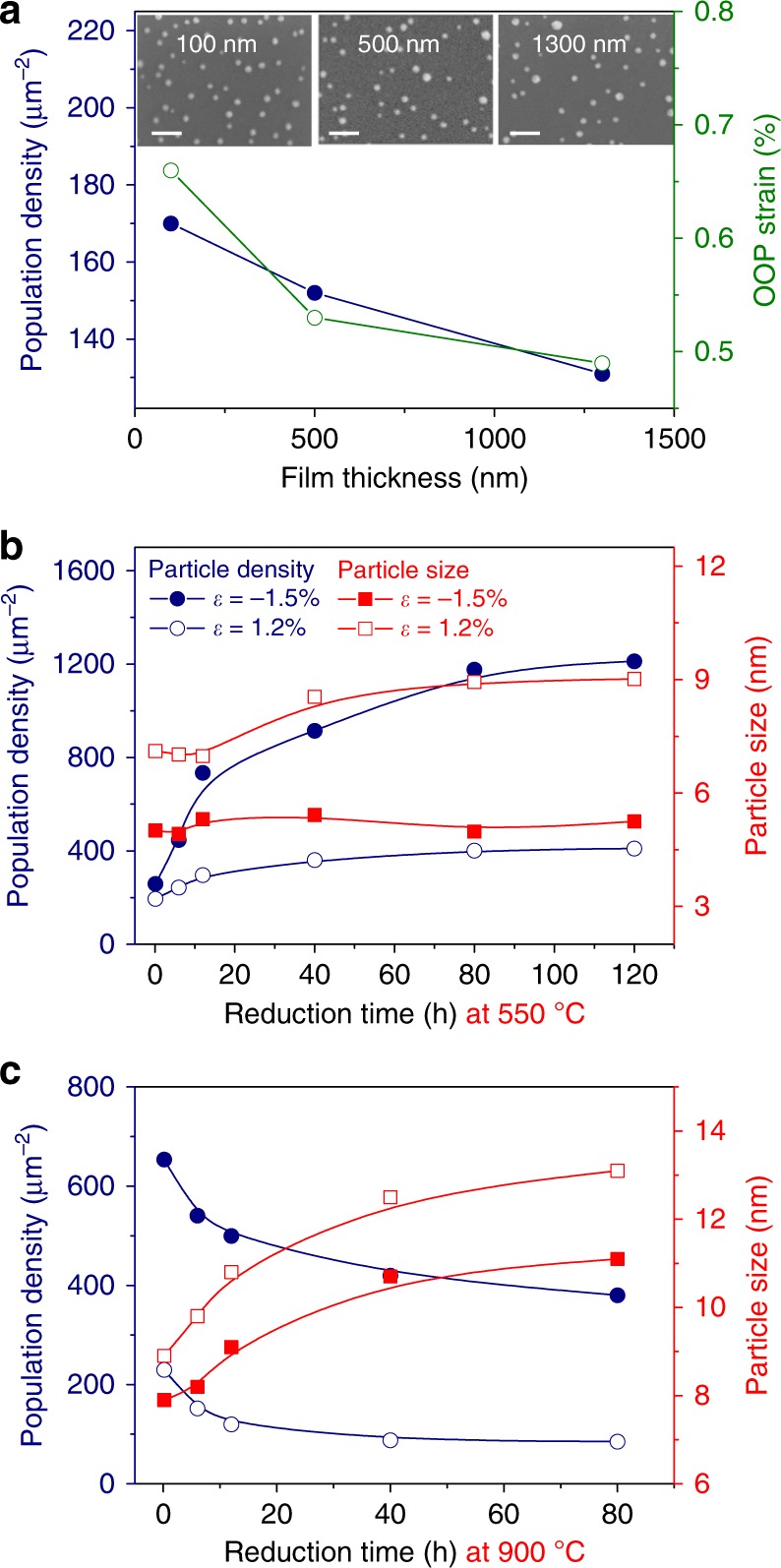
Thickness- and time-dependent exsolution. **a** Population density of exsolved particles and out-of-plane (OOP) strain of the LSNT film grown on the STO substrate as a function of the film thickness after the reduction at 900 °C for 12 h. Population density and particle size as a function of the reduction time (10 min, 6 h, 12 h, 40 h, 80 h, and 120 h) at **b** 550 °C and **c** 900 °C, respectively, for *ε* = −1.5% (LSNT/LSAT) and *ε* = +1.2% (LSNT/GSO) films

The degree of lattice strain can also be controlled by adjusting other parameters such as the film thickness (Fig. 3a and Supplementary Figure 5). As the film thickness of the LSNT/STO heterostructure increases from 100 to 1300 nm, the OOP tensile strain decreases due to relaxation of the strain. After the strained films are reduced (900 °C, 12 h), all the OOP lattice parameters decrease and reach nearly the same value. The thickness-dependent exsolution result reveals that the OOP strain can be well correlated with the population density of exsolved particles (Fig. 3a). The phenomena appear on all films and even on YSZ substrates (Supplementary Figure 5). It is interesting to note that the strain remains almost conformal even at a thickness as high as ~1.3 μm, as shown in the RSM data of Supplementary Figure 5a. This can be attributed to the structural softness involving octahedral rotation or tilting in perovskite oxides during growth^31, 32^, resulting in a remarkably high population density of exsolved particles even at μm-range thicknesses.

We have further investigated the tunability of exsolution in thin films by controlling the reducing conditions such as reduction temperature and time (Fig. 3b, c and Supplementary Figure 6). At 550 °C, the particle population density tends to increase gradually with the reduction time, but the particle size changes slightly (Fig. 3b). However, at 900 °C (Fig. 3c), the population density tends to decrease gradually with the reduction time while the particle size increases, indicating a coarsening process at a higher temperature. Notice that the LSNT/LSAT film with *ε* = −1.5% (“*ε* = −1.5% film” hereafter) reveals a fast exsolution with a population density of more than 500 particles μm^−2^ obtained in just 10 min at 900 °C, suggesting that the strain formed in the film is rapidly relaxed during the reduction, thereby accelerating the metal-particle exsolution.

### Thermodynamic and kinetic aspects of the strain-enhanced exsolution

The role of lattice strain in the exsolution process can be explained by exploiting thermodynamic and kinetic theories. The exsolution in a strained thin film can be described by the following three steps: (i) metal nucleation and growth, (ii) diffusion of nuclei to the surface, and (iii) coarsening of the exsolved particles.

At the beginning of the reduction process, Ni atoms exsolve from the oxide matrix and form nuclei. Classical nucleation theory can be used to account for the phenomena thermodynamically^33^. A nucleus stabilizes after it overcomes a critical nucleation barrier ($\Delta G_n^ \ast$). The total Gibbs free-energy change during the nucleation ($\Delta G_n$) is balanced by several factors; the surface/interface energy $(\gamma _{nc})$ and the bulk free energy ($\Delta G_b$) due to the bulk chemical-potential difference between the newly formed nucleus and the matrix phase. Because of the solid–solid phase transformation, the elastic strain energy ($\Delta G_s$) between the nucleus and the oxide matrix should be considered. Moreover, in the case of a strained film matrix (e.g., hetero-epitaxially grown film), we have to further consider the relaxation of the effective misfit-strain energy ($\Delta G_r$). For the nucleation/growth of a spherical particle with the radius *r*, $\Delta G_n$ can thus be written as

2 $$\Delta G_n = 4\pi r^2\gamma _{nc} + \frac{4}{3}\pi r^3\left( {\Delta G_b + \Delta G_s + \Delta G_r} \right)$$

The interface energy $({\mathrm{\gamma }}_{nc})$ and the elastic strain energy ($\Delta G_s$) are positive contributions, while both the bulk free energy ($\Delta G_b$) and the misfit-strain relaxation energy ($\Delta G_r$) are negative contributions for nucleation/growth. The critical nucleation barrier ($\Delta G_n^ \ast$) and the corresponding critical radius ($r^ \ast$) for the irreversible growth can be obtained by differentiating $\Delta G_n$ with respect to *r* and setting it to 0 ($\left. {\frac{{\mathrm{d}}\Delta {\mathrm{G}}_n}{{\mathrm{d}}r}} \right|_{r^ \ast } = 0$), resulting in

3 $$r^ \ast = - \frac{{2\gamma _{nc}}}{{(\Delta G_b + \Delta G_s + \Delta G_r)}}$$

4 $$\Delta G_n^ \ast = \frac{4}{3}\pi {\mathrm{\gamma }}_{nc}(r^ \ast )^2$$

Having obtained an expression of $\Delta G_n^ \ast$, one can further obtain an expression of the nucleation rate (${\mathrm{d}}N/{\mathrm{d}}t$) in terms of $\Delta G_n^ \ast$ using the Arrhenius-type kinetic equation:

5 $$\frac{{\mathrm{d}}N}{{\mathrm{d}}t} = A\;{\mathrm{exp}}\left( { - \frac{{\Delta {\mathrm{G}}_{n}^ \ast }}{{k_{B}T}}} \right)$$

where *N* is the number of particles formed, *A* is a pre-exponential factor, and *k*_*B*_ is the Boltzmann constant. According to the transition-state theory of rate processes^34^, *A* is not a constant but is given by $\frac{{k_BT}}{h}$, where *h* denotes the Planck constant.

From Eq. (3), it is clear that the critical radius ($r^ \ast$) for irreversible growth will decrease as the degree of misfit-strain relaxation ($\Delta G_r$) increases (i.e., more negative). In other words, the relaxation of the misfit strain tends to decrease the critical size of nuclei formed during the beginning stage of exsolution. Then, as can be deduced from Eq. (4), a decrease in the nucleation barrier is expected with the decrease in the size of the critical radius. According to Eq. (5), the relaxation of misfit strain also greatly expedites the production rate (d*N/*d*t*) of Ni nuclei from the oxide matrix owing to an exponential nature of Eq. (5). Thus, the misfit-strain energy can play a vital role in obtaining small sized metal nuclei with a high population density during exsolution.

Let us now examine the second step. When the nucleus grows beyond the critical size, the nucleus interacts with the surface leading to the formation of a pit. This diffusion step leads to the formation of embedded particles in a pit structure. A model of particle emergence from the bulk was proposed by Oh et al.^35^, although the following considerations could equally apply to other mechanisms also involving surface reorganization. The surface-energy term ($\gamma _{ex}$) is negative for the exsolution, in contrast to the nucleation step. On the other hand, the surface free energy due to pit formation (${\mathrm{\gamma }}_{pit}$) can be considered to be a positive term. In the exsolution of an unstrained bulk system, a favorable free-energy contribution to the particle emergence is the relaxation of the misfit-strain energy between the nucleus and the surrounding oxide matrix $(\Delta G_{nr} < 0)$. Thus, the nucleation at the surface can be determined from the interplay between the surface energy and the elastic strain energy^35–37^. Considering these three factors, one can write the net Gibbs free-energy change associated with the particle exsolution or emergence ($\Delta G_{ex}$) as

6 $$\Delta G_{ex} \propto 4\pi r^2\gamma _{ex} + \Delta A\gamma _{pit} + \frac{4}{3}\pi r^3(\Delta G_{nr})$$

where $\Delta A$ is the increase in the surface area due to the pit formation, and $r$ is the radius of the nucleus. In the case of a hetero-epitaxially strained film, one should include one additional term related to the relaxation of misfit-strain energy. This term ($\Delta G_{mr}$) is caused by the elastic relaxation of the lattice misfit-strain arising from the lattice mismatch between the oxide matrix and the substrate and this term further accelerates the particle emergence. Accordingly, two distinct elastic-strain terms contribute to $\Delta G_{ex}$ for a hetero-epitaxially strained film, and, in this case, Eq. (6) can be modified as

7 $$\Delta G_{ex} \propto 4\pi r^2\gamma _{ex} + \Delta A\gamma _{pit} + \frac{4}{3}\pi r^3(\Delta G_{nr} + \Delta G_{mr})$$

Since $\Delta G_{mr} < 0,$ the lattice misfit-strain term greatly expedites the diffusion of nuclei toward the surface. This consequently leads to a fast exsolution [${\mathrm{d}}N/{\mathrm{d}}t = {\mathrm{A}}e^{ - \Delta G_{\mathrm{ex}}^ \ast /{\mathrm{k}}_BT}$] and a small particle size or a high population density [$r^ \ast = - \frac{{2(\gamma _{ex} + k\gamma _{pit})}}{{(\Delta G_{nr} + \Delta G_{mr})}}$] of the exsolved particles, where we assume that $\Delta A = kr^2$ with *k* being a proportionality constant. As we have experimentally demonstrated (Fig. 3), the compressive-strained films have favorable structural stability during the reduction, as compared with the tensile-strained films. Thus, the compressive-strained film shows a higher degree of the strain relaxation (more negative $\Delta G_{mr}$), which results in a larger number of nanoparticles within a shorter reduction time (Fig. 3).

In the third final step, large particles grow at the expense of small ones, leading to particle coarsening. As shown in Fig. 3c, the size of nanoparticles increases with the reduction time, but the population density shows a reverse trend. It can be driven by the reduction of total energy because of the decrease in the overall surface-to-volume ratio, i.e., Ostwald ripening. This is a thermodynamic process governed by kinetic parameters such as atomic diffusion constants. The time evolution of isothermal particle coarsening can be described by a following self-limiting model form^38, 39^:

8 $$G(t) - G_0 = (G_{\mathrm{max}} - G_0)\left[ {1 - \exp \left( { - \frac{t}{\tau }} \right)} \right]$$

where *G*(*t*) is the average grain size at time *t*, *G*_0_ is the initial grain size, *G*_max_ is the limited grain size, and $\tau$ is the characteristic relaxation time (at 0.63 *G*_max_). Then, the diffusion coefficient (*D*) at a fixed temperature (*T*) can be written as

9 $$D = \frac{{\left( {G_{\mathrm{max}} - G_0} \right)^2}}{{4\tau }}$$

The surface diffusion coefficient of Ni at 900 °C for the compressive-strained films is $4.9 \times 10^{ - 23}$ m^2^ s^−1^, and that of the tensile-strained films is $8.1 \times 10^{ - 23}$ m^2^ s^−1^. These values are much smaller than that of the conventionally deposited Ni particles ($10^{ - 8} - 10^{ - 12}$m^2^ s^−1^ at 900–1000 °C)^40–42^. The estimated small atomic diffusion coefficient indicates slow particle coarsening and high resistance to the agglomeration.

**Fig. 4:**
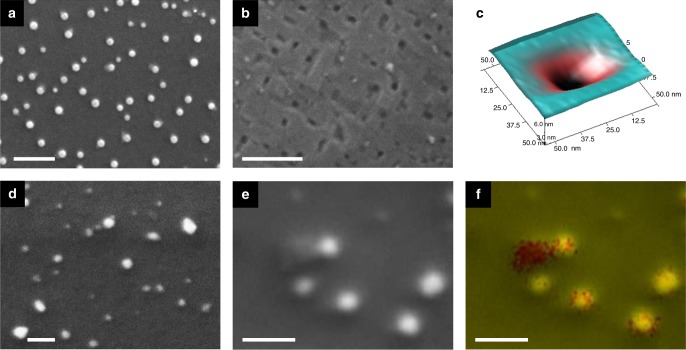
Thermal stability and coking resistance of the exsolved particles. SEM micrographs of **a** the reduced “*ε* = −1.5% film” (900 °C, 80 h), **b** after etching particles in HNO_3_, and **c** the corresponding 3D AFM images of pit. **d**, **e** SEM images after the coking test (pure CH_4_, 800 °C, 5 h), and **f** the corresponding EDS micrograph. Green color was used for the perovskite lattice, yellow for Ni metal, red for carbon. Scale bars, 100 nm

Figure 4 illustrates high resiliency to thermal coarsening and carbon coking in the strain-driven exsolved particles. We are able to etch the Ni particles in HNO_3_ for both compressive- and tensile-strained films after the reduction (900 °C for 80 h), resulting in many pits (Fig. 4a, b). The socketed structure of the 3D AFM profiles (Fig. 4c) implies a strong interaction between the Ni nanoparticles and the oxide surface, which leads to high thermal stability throughout the reduction process. In addition, we have examined the stability of exsolved particles to carbon coking in a hydrocarbon environment (pure CH_4_, 800 °C, 5 h) (Fig. 4d–f). Due to the strong interaction between the embedded metal particles and the oxide support, “base growth” of the carbon fiber proceeds without particle uplifting. All four thin films show this phenomenon of “base growth” rather than “tip growth”^12^, indicating a strong coking resistance of the exsolved Ni-nanoparticles. This interesting observation can be correlated with the smaller size of exsolved nanoparticles in thin films, as compared with bulk ceramic systems^12^. These are the main advantages of exsolved particles for high-temperature applications such as fuel/electrolysis cells, compared to conventionally deposited nanoparticles.

On the basis of thermodynamic and kinetic considerations, we deduce that the misfit-strain relaxation energy plays a significant role in the reduction process and leads to (i) a decrease in both nuclei size and nucleation barrier and (ii) an increase in the nucleation rate, resulting in a larger number of nuclei with a smaller particle size. The misfit-strain relaxation energy in a strained film ($\Delta G_{mr}$) can further promote the diffusion of nuclei to the surface, leading to a high population density of the exsolved nanoparticles. The extremely low diffusion coefficient during the coarsening process is attributed to the strong particle–perovskite oxide interaction of the embedded particle in a pit structure.

## Discussion

Epitaxial thin films show a strong correlation between lattice strain and exsolution of metal nanoparticles. Interestingly, the films maintain their single-crystallinity under highly reducing and high-temperature conditions. Because the film is strongly clamped to the substrate, the film undergoes a lattice contraction along the OOP direction. We judge that the unidirectional lattice change in strained thin films is accompanied by the relaxation of misfit-strain energy ($\Delta G_{mr}$) and consequently leads to a promotion of the nanoparticle nucleation with a large population density. This lattice strain-enhanced exsolution in thin films can be readily controlled by adopting various lattice mismatched substrates. It can be further controlled by adjusting deposition/reduction temperatures, film thickness, and so on. The deep submergence (~50%) of nanoparticles on the surface implies strong particle–oxide interaction and remarkable resistance to thermal agglomeration and carbon-coking problems. Furthermore, due to the robust strain effect on exsolution, the exsolution occurs well even at a low temperature of 550 °C, which is the target operating temperature of thin-film solid oxide fuel cells (SOFCs). The fairly vigorous particle-exsolution even at µm thicknesses represents suitable applicability to µ-SOFCs. This concept of the lattice strain-enhanced exsolution ($\Delta G_{mr} < 0$) can be potentially extended to other oxide and nano-composite systems. Moreover, the tunable nanoparticles can be applied to not only electrochemical devices but also nano-electronics, nano-magnetism, and nano-optics.

## Methods

### Thin-film fabrication

La_0.2_Sr_0.7_Ni_0.1_Ti_0.9_O_3−*δ*_ (LSNT) thin films were grown on various lattice mismatched substrates using PLD. These substrates include (i) (LaAlO_3_)_0.3_–(SrAl_0.5_Ta_0.5_O_3_)_0.7_ (LSAT) (001), (ii) SrTiO_3_ (STO) (001), (iii) DyScO_3_ (DSO) (110), and (iv) GdScO_3_ (GSO) (110). Oxygen partial pressure, deposition temperature, laser fluence, and repetition rate were fixed at 50 mTorr, 700 °C, 1.5 J cm^−2^, and 5 Hz, respectively. The film quality was determined by their crystallinity, uniformity, and smoothness. The LSNT target used for the PLD was calcined at 1300 °C, sintered at 1500 °C for 5 h in air by solid-state reaction method.

### Film reduction

The reduction of the deposited thin films was then performed in a controlled-atmosphere furnace with dry H_2_, at 550 and 900 °C, with the heating and cooling rates of 5 °C min^−1^. The coking test was carried out at 800 °C by flowing pure CH_4_ for 5 h.

### Characterizations

The structural analysis was performed based on symmetric XRD scans and RSM using a high-resolution X-ray diffractometer (D8 Discover, Bruker) under Cu Kα radiation operated at 40 kV and 40 mA. Microstructural and chemical information of thin films was obtained by using a field-emission scanning electron microscope (FE-SEM) equipped with an energy-dispersive X-ray (EDX) spectrometer (JSM-7800F PRIME, JEOL Ltd.). Atomic-scale structures of thin films were examined by employing high-resolution TEM method (JEM-ARM200F, JEOL with a Cs-corrector) under 200-kV acceleration voltage. Elemental composition and valence near the surface were measured using XPS (AXIS Ultra DLD, Kratos. Inc.), and the data were analyzed using XPSPEAK software. We have analyzed the Ni 3*p* peaks instead of Ni 2*p* peaks because the Ni 2*p*_3/2_ peak overlaps significantly with La 3*d*_3/2_ peak in the binding energy range of 850–860 eV. We used the Shirley background for the peak fitting. Surface morphology was examined by AFM (VEECO Dimension 3100). Particle analysis (size and density) was carried out using ImageJ software as shown in Supplementary Figure 7.

## Supplementary information

Supplementary Information

Supplementary Data 1

## Data Availability

The research data underpinning this publication can be accessed at 10.17630/21d12144-58ef-4f82-acd0-ba3c9a44ed72.
