# Author Correction: Lattice strain-enhanced exsolution of nanoparticles in thin films

**DOI:** 10.1038/s41467-019-10019-0

**Published:** 2019-05-02

**Authors:** Hyeon Han, Jucheol Park, Sang Yeol Nam, Kun Joong Kim, Gyeong Man Choi, Stuart S. P. Parkin, Hyun Myung Jang, John T. S. Irvine

**Affiliations:** 1 Department of Materials Science and Engineering, Pohang University of Science and Technology (POSTECH), Pohang, 37673 Republic of Korea; 2 Max Planck Institute of Microstructure Physics, Weinberg 2, Halle (Saale), 06120 Germany; 3 Gyeongbuk Science & Technology Promotion Center, Gumi Electronics & Information Technology Research Institute, Gumi, 39171 Republic of Korea; 4 Department of Materials Science and Engineering, Kumoh National Institute of Technology, Gumi, 39177 Republic of Korea; 5 1Fcell Inc., Pohang, 37673 Republic of Korea; 6 School of Chemistry, University of St Andrews, St Andrews, KY16 9ST Scotland UK; 7 Present Address: Department of Materials Science and Engineering, Massachusetts Institute of Technology, Cambridge, MA 02139 USA; 8 Present Address: Research Institute of Advanced Materials, Seoul National University, Seoul, 08826 Republic of Korea

**Keywords:** Catalyst synthesis, Electrocatalysis

Correction to: *Nature Communications* 10.1038/s41467-019-09395-4, published online 1 April 2019.

The original version of this Article contained an error in the Data Availability section, which incorrectly read ‘The data that support the findings of this study are available from the corresponding authors upon request.’ The correct version replaces this sentence with ‘The research data underpinning this publication can be accessed at 10.17630/21d12144-58ef-4f82-acd0-ba3c9a44ed72’. This has been corrected in both the PDF and HTML versions of the Article.

