# Supplementary material for: Lattice strain-enhanced exsolution of nanoparticles in thin films
Source: Nat Commun. 2019 Apr 1;10:1471. doi: 10.1038/s41467-019-09395-4 (PMC6443801; doi:10.1038/s41467-019-09395-4)
Supplement: Supplementary file 1 — Supplementary Information [file 41467_2019_9395_MOESM1_ESM.pdf]

Supplementary Information

Lattice Strain-Enhanced Exsolution of Nanoparticles in Thin Films

Han et al.

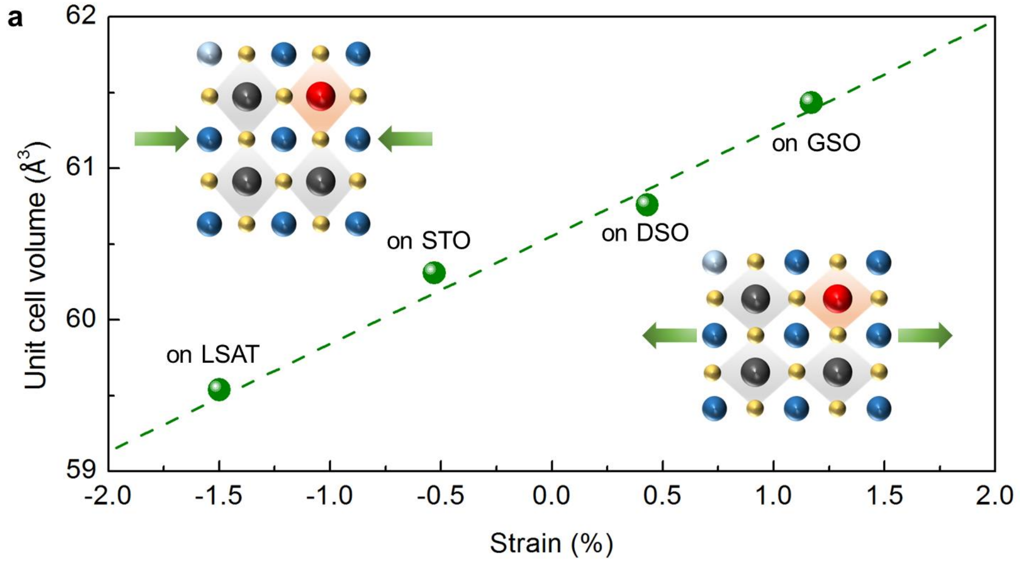

b

Substrate	Strain	Pristine film				Reduced film			
		a (Å)	c (Å)	V (Å^3)	c/a	a (Å)	c (Å)	V (Å^3)	c/a
LSAT	-1.5%	3.877	3.961	59.54	1.022	3.881	3.925	59.12	1.011
STO	-0.5%	3.906	3.953	60.31	1.012	3.906	3.92	59.81	1.004
DSO	+0.4%	3.942	3.91	60.76	0.992	3.941	3.884	60.32	0.986
GSO	+1.2%	3.972	3.894	61.43	0.980	3.968	3.878	61.06	0.977

Supplementary Figure 1. Strain analysis and structural parameters. (**a**) The unit-cell volume plotted as a function of the biaxial strain for as-grown (pristine) LSNT films on four different substrates. A green dashed line denotes the fitting result with the Poisson ratio of $\nu = 0.33$. According to the strain analysis, the evaluated lattice parameter of the unstrained LSNT film is 3.927 Å. (**b**) Structural parameters and the tetragonality (c/a) of pristine and reduced thin films on four different substrates.

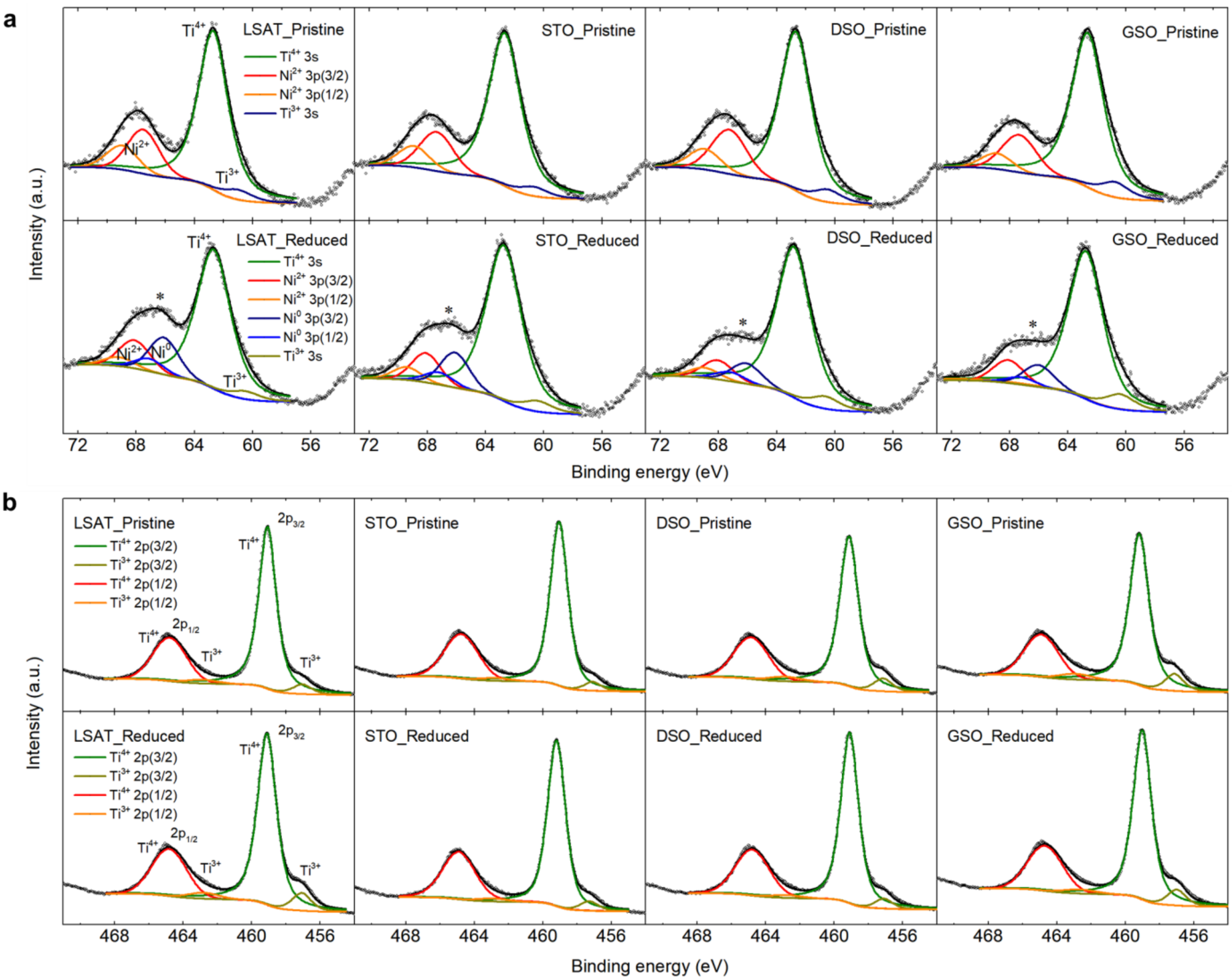

c

			LSAT (-1.5 %)	STO (-0.5 %)	DSO (+0.4 %)	GSO (+1.2 %)
Ni 3p	$Ni^0 / (Ni^0 + Ni^{2+})$ (%)	Pristine	0	0	0	0
		Reduced	56.2	53.4	50.3	48.9
Ti 3s	$Ti^{3+} / (Ti^{3+} + Ti^{4+})$ (%)	Pristine	5.6	5.4	7.1	8.4
		Reduced	4.8	5.7	6.7	8.3
Ti 2p	$Ti^{3+} / (Ti^{3+} + Ti^{4+})$ (%)	Pristine	6.0	5.1	7.7	9.1
		Reduced	6.0	5.5	5.4	8.5

Supplementary Figure 2. X-ray photoelectron spectroscopy (XPS) results. XPS spectra of (**a**) Ni 3p & Ti 3s and (b) Ti 2p peaks. (**c**) Quantification of the XPS spectra of the pristine and reduced LSNT films on four different substrates.

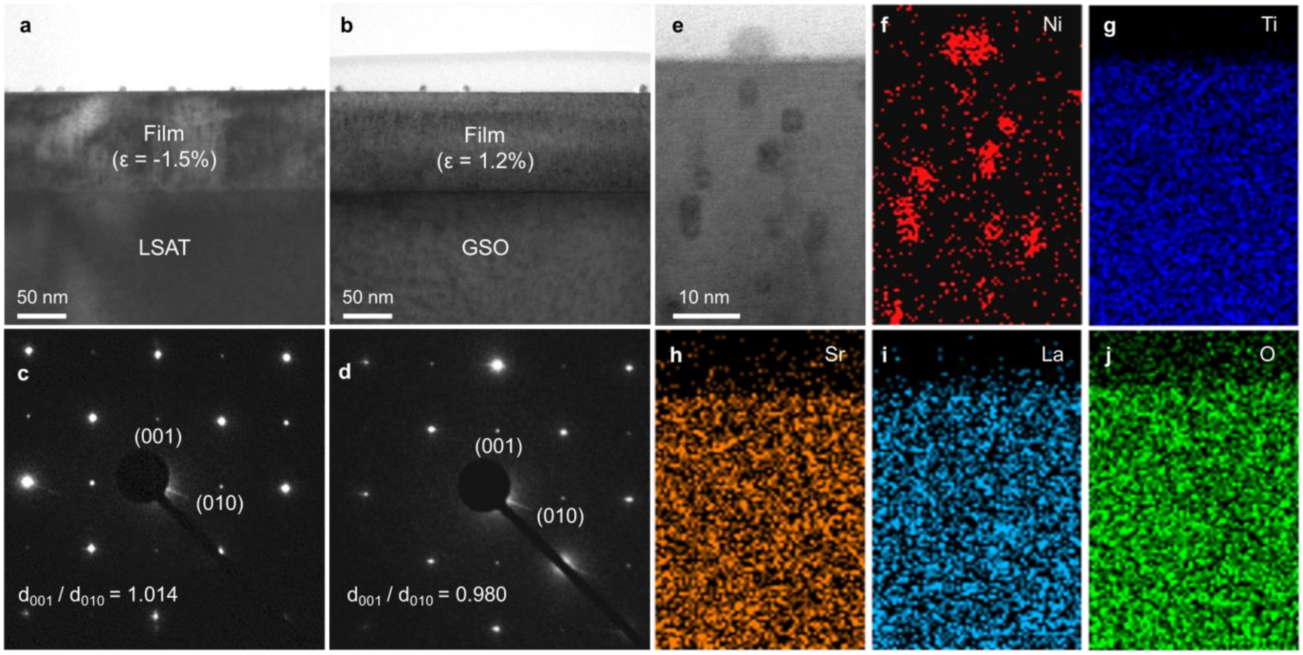

Supplementary Figure 3. *Ex-situ* HAADF STEM and corresponding EDS analysis (1). Cross-sectional HAADF STEM images of the *reduced* (dry H_2, **550 °C, 80 h**) LSNT films on (**a**) LSAT (ε = **–**1.5%) and (**b**) GSO (ε = +1.2%) substrates, and the corresponding SAED patterns of the LSNT films on (**c**) LSAT and (**d**) GSO substrates, respectively. The calculated ratio of the *d*-spacings in the SAED patterns does accord well with the estimated tetragonality (c/a) obtained from the XRD results (Fig. S1b). (**e**) Cross-sectional STEM image on the surface of the reduced (dry H_2, 550 °C, 80 h) LSNT film on LSAT (ε = -1.5%) and the corresponding EDS maps (**f-j**) of Ni, Ti, Sr, La, and O atoms, respectively. These STEM and EDS data not only show the exsolved particles on the surface but also demonstrate the Ni-cluster formation in the interior region.

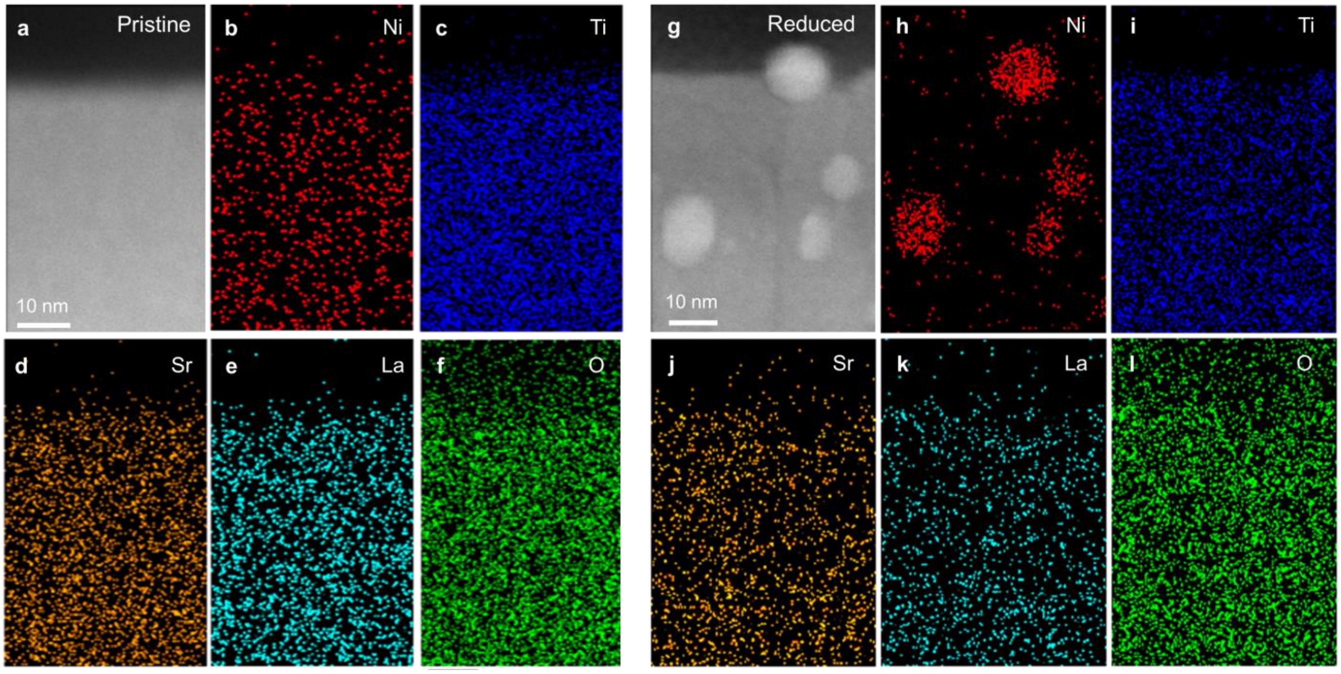

Supplementary Figure 4. *Ex-situ* STEM and corresponding EDS analysis (2). (**a**) Cross-sectional STEM image of the *pristine* LSNT film on GSO and the corresponding EDS maps (**b**-**f**) of Ni, Ti, Sr, La, and O atoms, respectively.

(**g**) Cross-sectional STEM image of the *reduced* (dry H_2, **900 °C, 12 h**) LSNT film on GSO and the corresponding EDS maps (**h**-**l**) of Ni, Ti, Sr, La, and O atoms, respectively. The pristine film shows a uniform distribution of Ni, while the reduced film clearly reveals Ni clusters within the film.

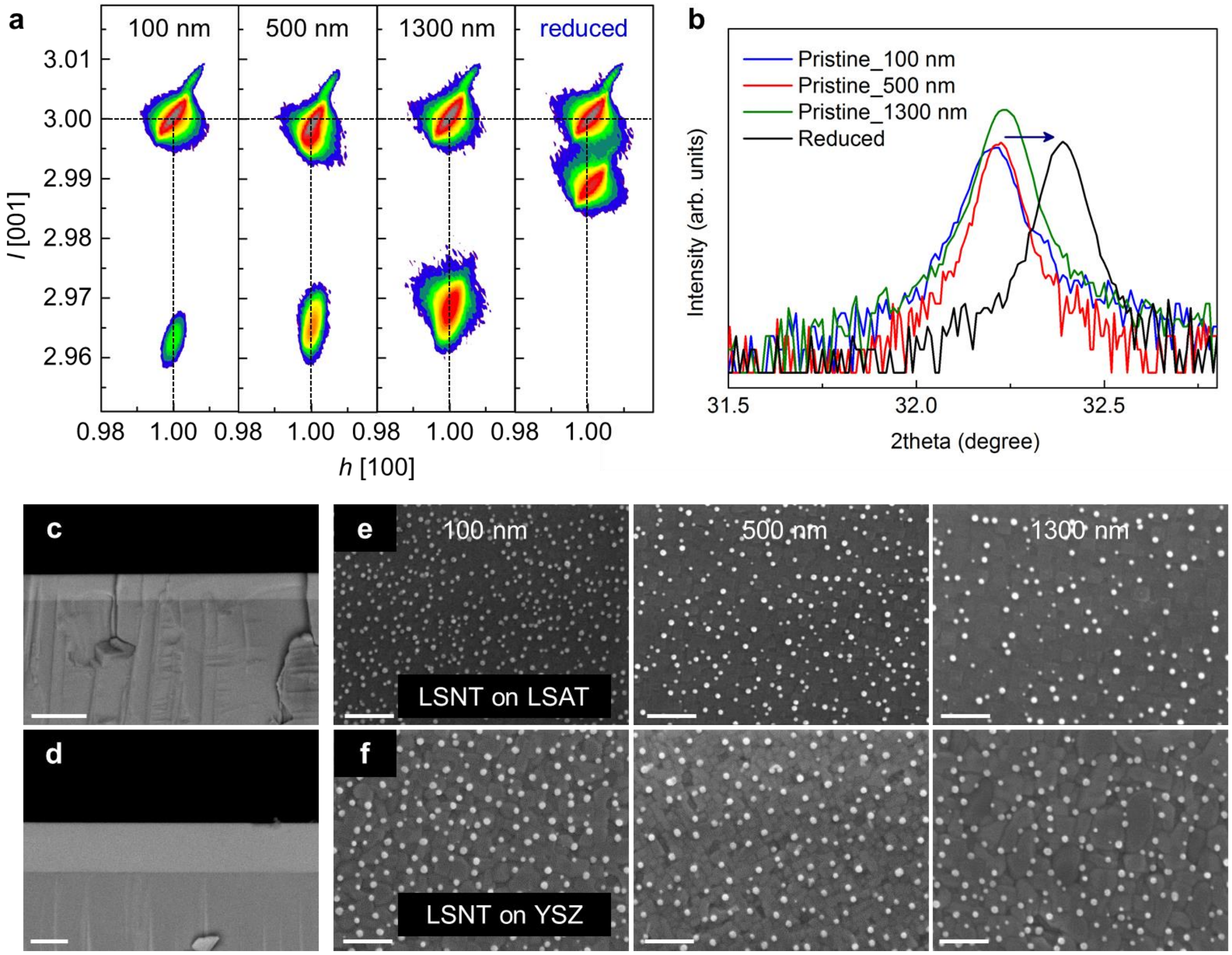

Supplementary Figure 5. Thickness-dependent exsolution (dry H_2, 900 °C, 12 h) in thin films. (**a**) Reciprocal space mapping (RSM) contours of pristine and reduced LSNT films on STO substrates with different film thicknesses. (**b**) θ-2θ XRD patterns of pristine and reduced LSNT (110) films on YSZ (001) substrates with different film thicknesses. Here, the reduced LSNT film has a thickness of 1300 nm. Cross-sectional SEM images of (**c**) 500-nm-thin and (**d**) 1300-nm-thin LSNT (110) films on STO substrates. Scale bars, 1000 nm. SEM images of the LSNT films grown on **(e)** LSAT (001) and **(f)** YSZ (001) substrates with different film thicknesses of 100 nm, 500 nm, and 1300 nm (from left to right). Scale bars, 200 nm.

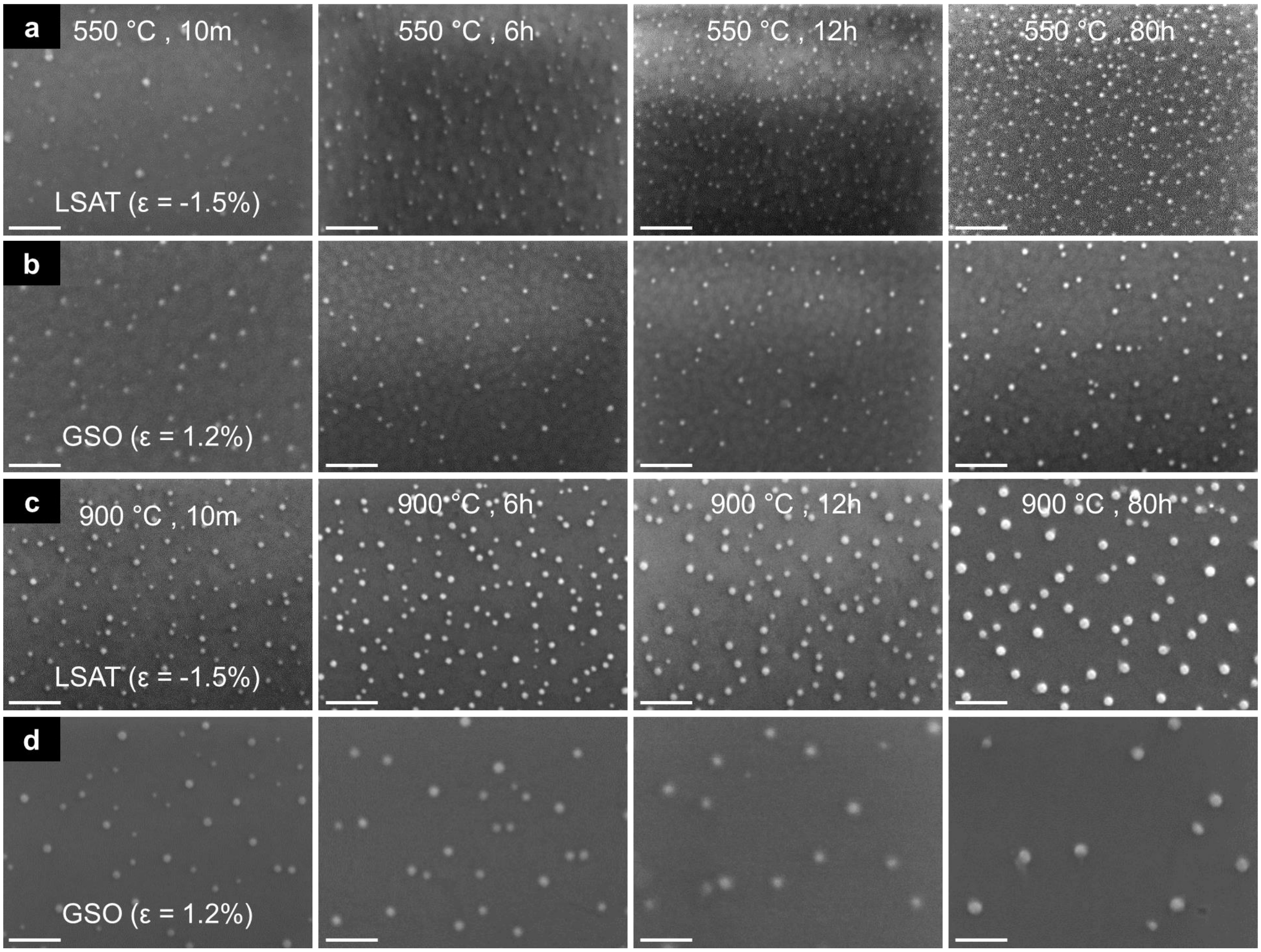

Supplementary Figure 6. SEM micrographs of the reduced LSNT films with different reduction conditions. SEM images of (**a**) ‘ε = –1.5% film’ on LSAT and (**b**) ‘ε = +1.2% film’ on GSO with different reduction times **at 550 °C**. SEM images of (**c**) ‘ε = –1.5% film’ and (**d**) ‘ε = +1.2% film’ with different reduction times **at 900 °C**. The four reduction times adopted in the experiment are 10 m, 6 h, 12 h, and 80 h (from left to right). Scale bar, 100 nm.

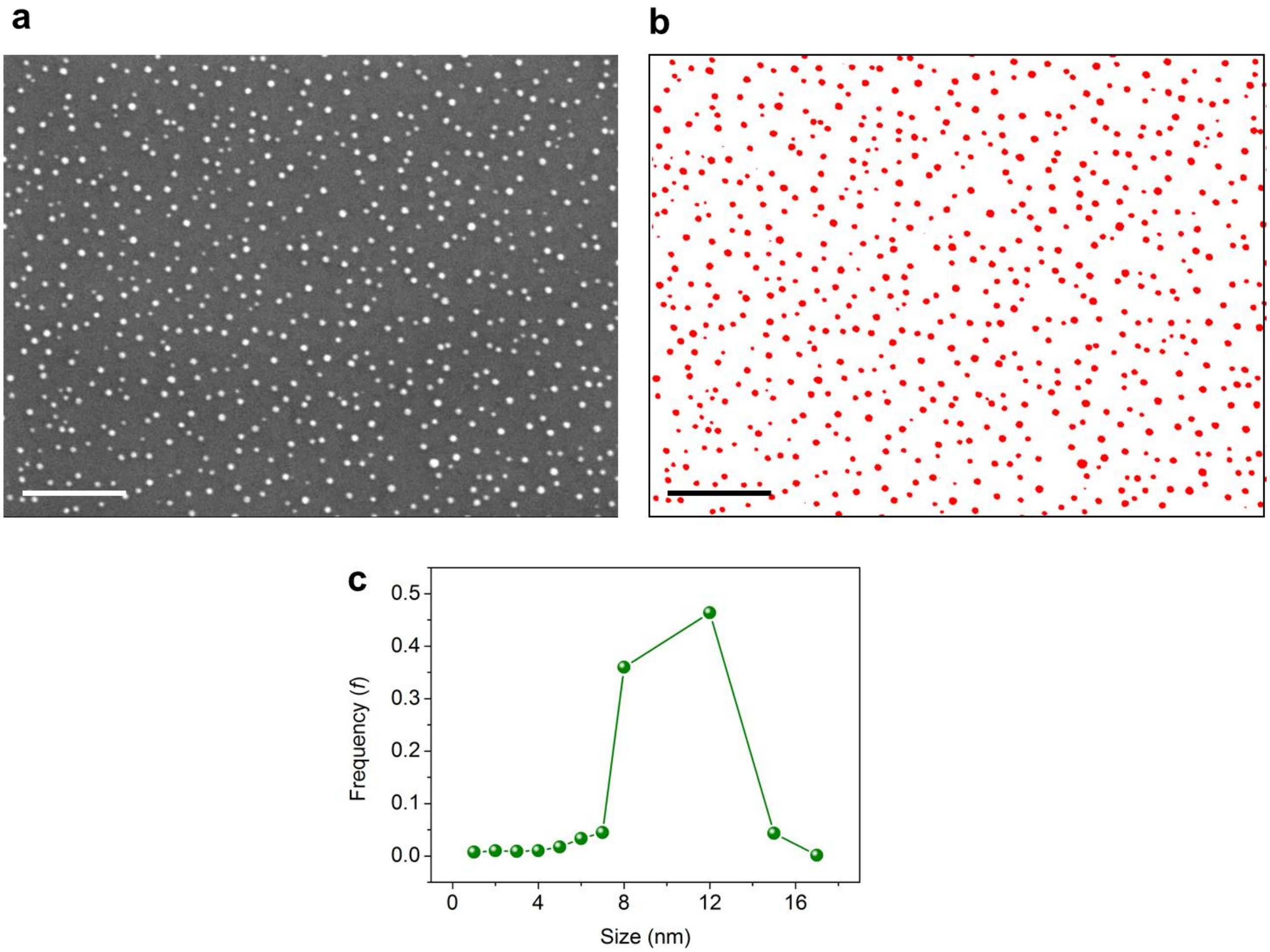

Supplementary Figure 7. Image and size of the exsolved particles on the surface of reduced LSNT film. (**a**) SEM image and (**b**) the corresponding particle mapping extracted by ImageJ of the reduced (900 °C, 10 m, dry H_2) '$\varepsilon = -1.5\%$ film' grown on a LSAT substrate. Scale bars, 200 nm. (**c**) Particle-size distribution profile obtained from ImageJ.
